# Sex-dependent effects of chronic intermittent hypoxia: implication for obstructive sleep apnea

**DOI:** 10.1186/s13293-024-00613-3

**Published:** 2024-04-25

**Authors:** Steve Mabry, Jessica L. Bradshaw, Jennifer J. Gardner, E. Nicole Wilson, Rebecca L. Cunningham

**Affiliations:** Department of Pharmaceutical Sciences, System College of Pharmacy, University of North Texas Health Science Center, 3500 Camp Bowie Boulevard, Fort Worth, TX 76107-2699 USA

**Keywords:** Chronic intermittent hypoxia, Sex differences, Inflammation, Oxidative stress, MitoTEMPOL, Novel object, Morris water maze, Open field, Marble burying

## Abstract

**Background:**

Obstructive sleep apnea (OSA) affects 10–26% of adults in the United States with known sex differences in prevalence and severity. OSA is characterized by elevated inflammation, oxidative stress (OS), and cognitive dysfunction. However, there is a paucity of data regarding the role of sex in the OSA phenotype. Prior findings suggest women exhibit different OSA phenotypes than men, which could result in under-reported OSA prevalence in women. To examine the relationship between OSA and sex, we used chronic intermittent hypoxia (CIH) to model OSA in rats. We hypothesized that CIH would produce sex-dependent phenotypes of inflammation, OS, and cognitive dysfunction, and these sex differences would be dependent on mitochondrial oxidative stress (mtOS).

**Methods:**

Adult male and female Sprague Dawley rats were exposed to CIH or normoxia for 14 days to examine the impact of sex on CIH-associated circulating inflammation (IL-1β, IL-6, IL-10, TNF-α), circulating steroid hormones, circulating OS, and behavior (recollective and spatial memory; gross and fine motor function; anxiety-like behaviors; and compulsive behaviors). Rats were implanted with osmotic minipumps containing either a mitochondria-targeting antioxidant (MitoTEMPOL) or saline vehicle 1 week prior to CIH initiation to examine how inhibiting mtOS would affect the CIH phenotype.

**Results:**

Sex-specific differences in CIH-induced inflammation, OS, motor function, and compulsive behavior were observed. In female rats, CIH increased inflammation (plasma IL-6 and IL-6/IL-10 ratio) and impaired fine motor function. Conversely, CIH elevated circulating OS and compulsivity in males. These sex-dependent effects of CIH were blocked by inhibiting mtOS. Interestingly, CIH impaired recollective memory in both sexes but these effects were not mediated by mtOS. No effects of CIH were observed on spatial memory, gross motor function, or anxiety-like behavior, regardless of sex.

**Conclusions:**

Our results indicate that the impact of CIH is dependent on sex, such as an inflammatory response and OS response in females and males, respectively, that are mediated by mtOS. Interestingly, there was no effect of sex or mtOS in CIH-induced impairment of recollective memory. These results indicate that mtOS is involved in the sex differences observed in CIH, but a different mechanism underlies CIH-induced memory impairments.

**Supplementary Information:**

The online version contains supplementary material available at 10.1186/s13293-024-00613-3.

## Highlights

- This is the first study to examine the contribution of sex on CIH-induced inflammation, steroid hormones, oxidative stress, and behavioral dysfunction in rats.
- CIH increased circulating inflammation and fine motor dysfunction in females, whereas CIH increased circulating oxidative stress and compulsivity in males.
- Inhibition of mitochondrial oxidative stress blocked sex-specific effects of CIH in males and females.
- CIH induced recollective memory impairments in males and females, which was not prevented by blocking mitochondrial oxidative stress.

**Supplementary Information:**

The online version contains supplementary material available at 10.1186/s13293-024-00613-3.

## Plain language summary

Sleep apnea is a common sleeping condition in adults with a wide range of symptoms that include inflammation, oxidative stress, memory problems, anxiety, and compulsivity. Men are diagnosed with sleep apnea more often than women. Although there is limited information on how sleep apnea affects men and women differently, previous studies suggest that women may exhibit different sleep apnea symptoms than men. To examine the impact of male and female sex on common sleep apnea symptoms, we exposed adult male and female rats to a model of sleep apnea called chronic intermittent hypoxia (CIH). We found that many effects of CIH were different in males and females. CIH females had increased inflammation and motor problems, whereas CIH males had increased oxidative stress and compulsivity. To investigate the reason for these CIH sex differences, we blocked mitochondrial oxidative stress. Blocking mitochondrial oxidative stress decreased CIH associated sex differences. However, blocking mitochondrial oxidative stress had no impact on CIH-induced memory impairment that was observed in male and female rats. Our findings support previous reports that suggest that women exhibit different sleep apnea symptoms than men. Further, we extend these findings by showing that mitochondrial oxidative stress is involved in these sex differences. Clinically, patients diagnosed with sleep apnea are typically treated with continuous positive airway pressure (CPAP) machines, which have high rates of non-compliance (15–40%). Therefore, understanding why sleep apnea is causing these symptoms will be important in developing therapeutics.

**Supplementary Information:**

The online version contains supplementary material available at 10.1186/s13293-024-00613-3.

## Background

Obstructive sleep apnea (OSA) is a highly prevalent sleeping disorder which is observed to affect between 10 and 26% of adults [1–3]. Men are 2–4 times more likely to be diagnosed with OSA than women [1–3]. However, the prevalence of OSA may be under-reported for women, as OSA symptoms are different in women compared to men [4]. Women are more likely to present with atypical symptoms of OSA, even in mild OSA, that is associated with lower levels of apnea–hypopnea index (AHI) scores (hypoxic events per hour of sleeping) than men [4, 5]. Symptoms of OSA include cognitive dysfunction, anxiety, and compulsivity [6–12], along with increased inflammation and oxidative stress (OS) [13–18], decreased testosterone in men [19–21], and decreased estradiol and progesterone in women [22–24]. Though women have been observed to have greater risk for OSA-associated cognitive dysfunction [3, 25], the role of sex on OSA symptoms is not completely understood.

Patients diagnosed with OSA are typically treated with continuous positive airway pressure (CPAP) machines, the gold standard for therapy [26–28]. Effective CPAP therapy can reduce inflammation, OS, and cognitive dysfunction [27, 29–31]. However, adherence to CPAP therapy is a major issue, as 15–40% of patients are non-compliant with prescribed machine usage [26–28]. Even patients that are adherent to CPAP therapy may not be receiving proper treatment, due to anatomical variations or ineffective machine air pressure titration [26, 28]. This lack of efficacy has led to increased research to determine OSA mechanisms with the goal of developing conjunctive pharmaceutical therapies for OSA [32–34]. Current drugs that are approved for OSA treatment are focused on symptom therapy, such as reducing daytime sleepiness and improving attention [33, 34] rather than OSA pathophysiology as a result of OSA hypoxia.

Hypoxia induced by OSA is observed to increase many circulating markers of OS and inflammation [13–18]. This can induce a feed-forward cycle, in which chronic inflammation increases OS [35, 36], and chronic OS can increase inflammation [37, 38]. In addition, sex also plays a role in OS and inflammation [39, 40]. Women exhibit higher baseline levels of OS compared to men [41–43]. Furthermore, women experience greater levels of inflammation and autoimmunity compared to men [44–46]. Based on these findings, sex differences observed in OSA may be related to basal sex differences in inflammatory and OS status. Since the mitochondria are one of the primary generators of OS within the cell [47, 48] and mitochondrial dysfunction has been observed in OSA [49–51], mitochondrial OS (mtOS) may mediate sex differences in OSA.

To examine the impact of sex on OSA-associated symptoms (inflammation, OS, memory, motor, anxiety, compulsivity), we utilized an experimental rat model of OSA called chronic intermittent hypoxia (CIH). CIH exposure replicates the fragmented episodes of hypoxia observed in OSA [52]. Further, to examine the mechanisms of mtOS on sex differences in CIH, we implanted osmotic pumps subcutaneously in a subgroup of rats to continuously administer a mitochondria-targeting superoxide dismutase mimetic, MitoTEMPOL (MT), to block mtOS. The inflammatory, OS, and behavioral phenotypes of CIH in males are clearly defined. CIH increases inflammation [6, 53, 54], increases OS [6, 55, 56], impairs recollective memory [57–59], impairs spatial learning and memory [53, 60–62], and increases anxiety-like behavior [57, 58, 63, 64]. Additionally, we have previously reported that CIH reduced testosterone and did not affect corticosterone in young adult male rats [65]. Studies examining sex as a biological variable in CIH are scarce. Most of these studies have been conducted only in mice, showing CIH increased circulating OS in male but not female mice [55] and impaired spatial learning and memory in ovariectomized female mice but not intact females [66]. Given the limited data on the effects of CIH in females, we predicted that CIH would produce sex-dependent increases in inflammation and OS, alongside cognitive dysfunction. Further, we predicted that inhibiting mtOS would prevent the CIH phenotype of increased inflammation, increased OS, and cognitive dysfunction.

## Methods

### Animals

All experiments were conducted using adult virgin Sprague Dawley male and female rats (aged 3–4 months, Charles River, Wilmington, MA). Male and female rats were housed in separate rooms in our animal facility on a 12-h (hr) reverse light cycle (lights were off at 09:00). Reverse lighting allowed behavioral testing to be conducted during the active phase of the circadian cycle. Food and water were provided ad libitum. All rats were randomly assigned to either vehicle or MT, and either normoxia (room air) or CIH treatment conditions. Our original groups were planned to be 6–8 rats per group. However, due to unforeseen circumstances (i.e., severe winter storms in Texas) that terminated the CIH software protocol that intermittently decreases oxygen levels in the chambers prior to collection of plasma, additional cohorts of animals were included to collect sufficient plasma for analysis. All cohorts were behaviorally tested to allow for consistent experimental protocols in the plasma cohorts. The following groups sizes reflect the maximum number of rats per group that were analyzed. Female: Normoxic Vehicle (n = 10), Normoxic MT (n = 6), CIH Vehicle (n = 10), CIH MT (n = 10); Male: Normoxic Vehicle (n = 13), Normoxic MT (n = 9), CIH Vehicle (n = 7), CIH MT (n = 7). To acclimatize the rats to operator handling and reduce stress responses during behavior testing, rats were handled daily, beginning one week prior to the start of behavior testing. At the conclusion of behavior testing, the rats were anesthetized with 2–3% isoflurane and euthanized via decapitation during the active phase of the circadian cycle (09:00–11:00). All experiments were conducted in agreement with the Guide for the Care and Use of Laboratory Animals of the National Institutes of Health and the ARRIVE guidelines. These protocols were approved by the Institutional Animal Care and Use Committee of the University of North Texas Health Science Center.

### Osmotic minipump implantation

One week prior to the initiation of CIH, all rats were instrumented with an osmotic minipump (Alzet Mini-Osmotic Pump Model 2002, Durect Corporation, Cupertino, CA) implanted subcutaneously between the scapula [67]. Minipumps contained either 0.9% saline vehicle or MitoTEMPOL (0.7 mg/kg/day; Caymen Chemical Company, Ann Arbor, MI; MT) dissolved in saline. MT combines the antioxidant moiety TEMPOL, with a lipophilic cation triphenylphosphonium [68, 69]. Triphenylphosphonium increases mitochondrial aggregation of MT by several 100-fold over TEMPOL alone [69–71]. MT has been observed to reduce mtOS in vitro and in vivo [71, 72]. Drugs like MT are easily administered with multiple biologically active routes of administration and high blood–brain barrier permeability [73–75]. The dose of MT was chosen based on previous in vivo studies [72, 76, 77]. To ensure proper osmotic function as indicated by bubbling present on the surface of pumps, all pumps were incubated in a 37 °C water bath for at least 24 h prior to implantation. Surgeries were performed using aseptic techniques with isoflurane (2–3%) anesthetic.

### Chronic intermittent hypoxia protocol

One week prior to the initiation of the CIH protocol, the home cages (clear plastic containers) were placed into Oxycycler chambers (76.2 × 50.8 × 50.8 cm, BioSpherix, Lacona, NY, USA) to acclimatize the rats. During their sleep phase of the circadian cycle, CIH was performed for 8 h starting at 21:00. The CIH protocol consisted of intermittent oxygen reduction from 21% (room air) to 10% in 6-min cycles per hour (i.e., 10 cycles/h) over 8 h/day for a period of 14 days, as previously described [6, 52, 78, 79]. 10 CIH cycles per hour results in an apnea–hypopnea index (AHI) of 10, which is consistent with mild sleep apnea in humans [31, 79].

### Behavioral tasks

Behavioral studies were conducted between days 8 and 14 of CIH from 09:45 to 17:00 during the active phase of the circadian cycle. The order of the behavior tests was randomized. Male and female rats were behaviorally tested in separate cohorts to prevent potential confounding effects of pheromones on behavior [80, 81]. All testing equipment (e.g., marbles, arenas, tanks) were thoroughly cleaned with 70% ethanol between each rat. All behavior studies were conducted under red lighting and recorded for later analysis by an investigator blinded to treatment groups. Behavior tests were used to assess compulsive behaviors (marble burying test) [82, 83], fine motor function (footfalls, rearing behavior) [56], anxiety-like behaviors (center entries and center duration in an open field) [84, 85], spatial learning and memory (Morris water maze) [84, 86], and recollective memory (novel object) [87, 88]. Rats have been observed to exhibit learning in response to repeated testing in similar environments, which has been described as a test battery effect [89–91]. To avoid a learning confound due to battery testing (e.g., open field tests [89–91] and Morris water maze [89, 90]), one behavioral task per index of interest was performed [78].

### Modified open field: fine motor function

Fine motor function was assessed using a small novel open field arena (40.64 × 40.64 × 38.1 cm) with a bi-directional main field bar (San Diego Instruments Photobeam Activity System; Open Field Arena), as previously published [52, 56, 78]. To increase the difficulty of locomotion, a wire mesh platform elevated 2 cm above the floor was placed in the arena. The rats were allowed 10 min to explore the arena. Fine motor function was classified by distance traveled, rearing behavior (assisted, unassisted, total), and footfalls past the elevated wire mesh. Individual rears were classified as either assisted or unassisted. These were defined by whether the animal reared with (assisted) or without (unassisted) its forelimbs braced against the wall [52].

### Open field: gross motor function and anxiety-like behavior

Using a large novel open field arena (60.96 × 60.9 × 38.1 cm), we assessed gross motor function and anxiety-like behavior during a 5-min trial. Behaviors were recorded using ANY-maze software (v. 5.14, Stoelting CO.). This open field duration has been previously observed to be sensitive to differences in anxiety-like behavior and stress in rats [92–94]. Gross motor function was examined by distance traveled in the open field. Anxiety-like behaviors were tracked by number of entries (frequency) to the center of the field and time spent (duration) within the center of the open field [56, 78, 95]. In addition, this task allowed habituation to the large open field arena in order to conduct the novel object recognition task.

### Novel object recognition task: recollective memory

Following habituation to the open field test using the large arena (60.96 × 60.9 × 38.1 cm), we conducted the novel object recognition task [56]. In this task, two identical objects (building blocks) were placed in adjacent corners of the arena, and the rats were given 5 min to interact with the objects and then removed from the arena. One hour later, one of the objects was replaced with a novel object (spherical toy ball), and the rats were given 3 min to explore the arena and interact with the objects. Contacts with the novel object and latency to the novel object were recorded as measures of short-term recollective memory retention [56, 87, 88]. Rats that only engaged with the familiar object were marked as performing zero contacts at the maximum test latency (180 s).

### Morris water maze: spatial learning and memory

To examine spatial memory, the Morris water maze test was used according to our published protocols [56, 78]. Behaviors were recorded using ANY-maze software (v. 5.14, Stoelting Co.). Over a period of 4 days, rats were trained to find a submerged platform in a pool filled with opaque water (23–25 °C) and remain on the platform for 20 s until removed by the operator. Day 1 of training consisted of visible platform pre-training where the platform was moved on each trial (3/day). On days 2–4, the platform was submerged for spatial training and remained in the same position for all trials. A learning index (LI) was generated using the latency data from days 2, 3, and 4 of spatial training. The LI was generated as the sum total of the average latency to the target of all trials in blocked means for each training day [78, 96]. The LI scores were used as indicators of spatial learning where lower LI scores indicated greater spatial learning ability [96, 97]. Spatial memory was assessed following 4 days of training, in which each rat was administered a probe trial (the underwater platform was removed). Latency and pathlength to the target during the probe trial were used as indicators of spatial memory.

### Marble burying test: compulsive behavior

To examine compulsive behaviors, the marble burying test was conducted following published protocols [78, 82, 83]. To conduct this test, the floor of the testing arena (50 × 25 × 30 cm) was thoroughly covered with 1 cm of rodent bedding litter to allow the rats to easily bury marbles. Twenty marbles of similar color and size (1.5 cm) were spaced evenly in a 4 × 5 grid on one side of the arena base. Each rat was given 10 min to explore and interact with the marbles. Operators visually monitored the experiment and manually recorded behaviors. After 10 min, the rat was removed and the number of marbles buried (75% or more) was photographed and quantified.

### Sample collection

At the conclusion of behavior tests, rats were anesthetized with isoflurane (2–3%) and decapitated during the first 2 h of the rats’ active phase of the circadian rhythm to collect tissue and plasma samples [6, 52, 56, 78]. Trunk blood was collected in EDTA tubes, and then centrifuged at 2000 × g for 10 min at 4 °C to collect plasma. Plasma was stored at − 80 °C until assayed for circulating inflammation and oxidized proteins.

### High throughput multiplex: inflammation panel

A MILLIPLEX^®^ rat cytokine/chemokine magnetic bead panel (Sigma Millipore, Cat # RECYTMAG-65 K) utilizing antibodies against IL-1β, IL-6, IL-10, and TNF-α was used to quantify circulating inflammatory cytokines. IL-6, TNF-α, and IL-1β were selected as common pro-inflammatory cytokines [98–100], while IL-10 was selected as a common anti-inflammatory cytokine [101]. All samples were diluted 1:2 in assay buffer prior to running the assay according to manufacturer’s instructions. Samples were run in duplicate and cytokines were measured on Luminex^®^ 200™ using xPONENT^®^ software version 4.3 (Luminex Corporation, Austin, TX). Quality control values for each cytokine were within the range provided by the manufacturer. The ratio of IL-6/IL-10 was used to investigate the overall relationship between pro- and anti-inflammatory cytokines [102].

### High throughput multiplex: steroid hormone panel

A MILLIPLEX^®^ multi-species hormone magnetic bead panel (Sigma Millipore, Cat # MSHMAG-21 K) utilizing antibodies against corticosterone, estradiol, testosterone, and progesterone were used to quantify circulating steroid hormones. Steroid hormones were extracted from plasma via acetonitrile preparation as instructed by the manufacturer and previously published [78, 103]. Briefly, 150 µl of plasma was diluted in 225 µl acetonitrile, vortexed, and incubated at room temperature for 10 min. Samples were then centrifuged at 17,000 × *g* for 5 min at 4 °C*.* Supernatants were dried via vacuum centrifugation, and pellets were reconstituted in 120 µl assay buffer prior to analysis. Samples were run in duplicate and an 18-h overnight incubation was performed according to manufacturer instructions. Hormones were measured on a Luminex^®^ 200™ instrument using xPONENT^®^ software version 4.3 (Luminex Corporation, Austin, TX). Quality control values for each hormone were within the range provided by the manufacturer.

### Advanced oxidation protein products assay

Circulating plasma OS was assayed using OxiSelect Advanced Oxidation Protein Products (AOPP) kit (Cell Biolabs, Inc., San Diego, CA) according to our previously published protocols [6, 52]. The micromolar (µM) concentration of oxidized proteins in the plasma were measured relative to a known standard. Chloramine in the kit reacts with oxidized proteins to produce a color change, which is read at 340 nm. To account for colorimetric interference from the plasma samples, a background correction was performed for all samples. All samples were diluted 1:2 in assay buffer for analysis.

### Statistical analysis

Statistical analyses were conducted in IBM^®^ SPSS^®^ (SPSS^®^ v. 29.0.0, IBM^®^, 2022). Normality of data distribution was tested using the Shapiro–Wilk test. Data with non-Gaussian distribution were normalized by log base 10 transformation (x = lg10(x)) or by square root transformation (x = sqrt(x)) through the SPSS^®^ transformation function. All data which were normalized for analysis are identified as “transformed” in the respective figure or table legend. Outliers greater than 2 standard deviations from the mean were removed from analysis. 3-way ANOVAs were conducted using the factors of CIH, drug treatment, and sex. For all analyses, we provide the F values, degrees of freedom, p-values, and η^2^ (measure of effect size). Following ANOVA testing, a Fisher’s LSD post-hoc test was used to determine specific group differences for significant results. Results are presented as mean ± S.E.M. unless otherwise indicated. Significance was defined as p ≤ 0.05. Post-hoc significance is indicated in figures and tables by unique letters (p ≤ 0.05). Individual letters reflect sets of data which are not significantly different from each other. For example, if two bars within a graph have the letter ‘a’, then these two groups are not significantly different from each other. All figures in which multiple letters are presented together (e.g., abc) refer to unique comparisons of a, b, and c, respectively. All post-hoc comparisons are unique to the specific figure or panel within the figure.

## Results

### CIH induced a pro-inflammatory state in female rats that was reduced by blocking mitochondrial oxidative stress

**Fig. 1:**
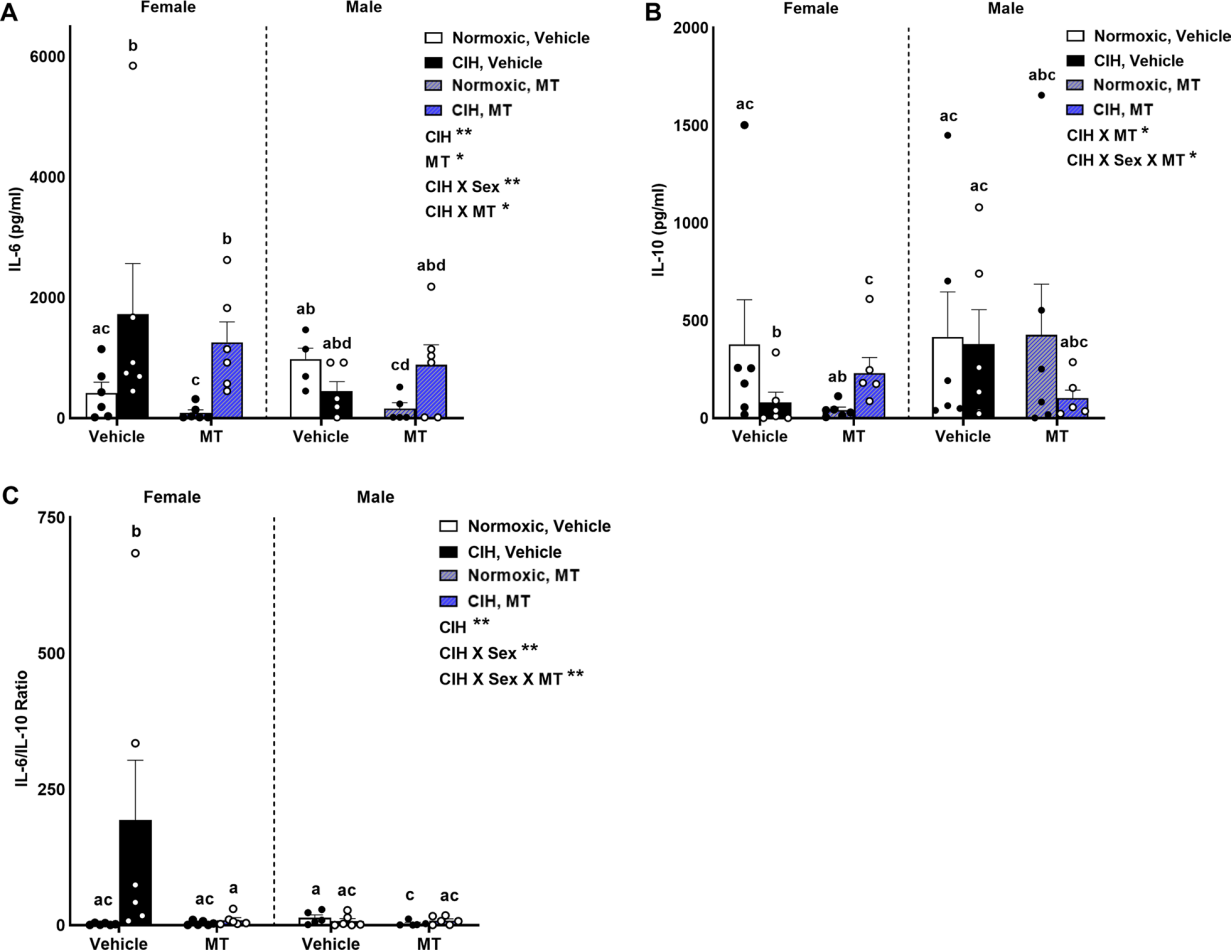
Relationship between pro- and anti-inflammatory cytokines. CIH shifted females to a pro-inflammatory state. CIH specifically elevated IL-6 levels in females, which was not affected by MT (**A**). MT lowered IL-6 levels in normoxic but not CIH rats (**A**). Vehicle female rats exposed to CIH had lower IL-10 levels (**B**). In CIH females, MT elevated IL-10 levels (**B**). The IL-6/IL-10 ratio was only significantly elevated in vehicle CIH females (**C**). In males, MT lowered the IL-6/IL-10 ratio only in the normoxic rats (**C**). Data for inflammatory cytokines was log transformed for analysis. Raw values are shown and error bars denote mean ± S.E.M. Analyzed by 3-way ANOVA with Fisher’s LSD multiple comparisons tests. ANOVA significance indicated by: ***p < 0.001; Post-hoc significance indicated by unique letters (p ≤ 0.05). Significant effects observed (**A**): CIH (F_1, 38_ = 10.123; p = 0.003; η^2^ = 0.146); MT (F_1, 38_ = 6.055; p = 0.019; η^2^ = 0.087); CIH X Sex (F_1, 38_ = 7.369; p = 0.010; η^2^ = 0.106); (F_1, 38_ = 6.348; p = 0.016; η^2^ = 0.091). Significant effects observed (**B**): CIH X MT (F_1, 40_ = 4.437; p = 0.041; η^2^ = 0.083); CIH X Sex X MT (F_1, 40_ = 5.412; p = 0.025; η^2^ = 0.101). Significant effects observed (**C**): CIH (F_1, 38_ = 7.403; p = 0.010; η^2^ = 0.110); CIH X Sex (F_1, 38_ = 7.334; p = 0.010; η^2^ = 0.109); CIH X Sex X MT (F_1, 38_ = 9.344; p = 0.004; η^2^ = 0.139). Individual letters reflect sets of data which are not significantly different from each other. Should two bars within a graph have the same letter, then these two groups are not significantly different from each other. All figures in which multiple letters are presented together (e.g., abc) refer to unique comparisons of a, b, and c respectively. *CIH* chronic intermittent hypoxia, *MT* MitoTEMPOL

Levels of circulating IL-6 were dependent on CIH (p = 0.003) and inhibition of mtOS (p = 0.019; Fig. 1A). In addition, significant interactions between CIH and sex (p = 0.010) and CIH and MT (p = 0.016) were observed on IL-6 levels (Fig. 1A). CIH specifically elevated IL-6 levels in females (p ≤ 0.05), which was not affected by MT (Fig. 1A). MT lowered IL-6 levels in normoxic rats but not CIH rats (p ≤ 0.05). IL-10 levels were also dependent on CIH, sex, and inhibition of mtOS with MT. Significant interactions between CIH and MT (p = 0.041) and CIH, sex, and MT (p = 0.025) were observed on IL-10 levels (Fig. 1B). These interactions were observed only in the female rats. Vehicle female rats exposed to CIH had lower IL-10 levels (p ≤ 0.05). In CIH females, MT elevated IL-10 levels (p ≤ 0.05). When comparing IL-6 to IL-10 levels, the ratio was dependent on CIH (p = 0.010), the interaction of CIH and sex (p = 0.010) and the interaction of CIH, sex and inhibition of mtOS (p = 0.004; Fig. 1C). The IL-6/IL-10 ratio was only significantly elevated in vehicle CIH females (p ≤ 0.05). In males, MT lowered the IL-6/IL-10 ratio, though this was only in the normoxic rats (p ≤ 0.05).

**Fig. 2:**
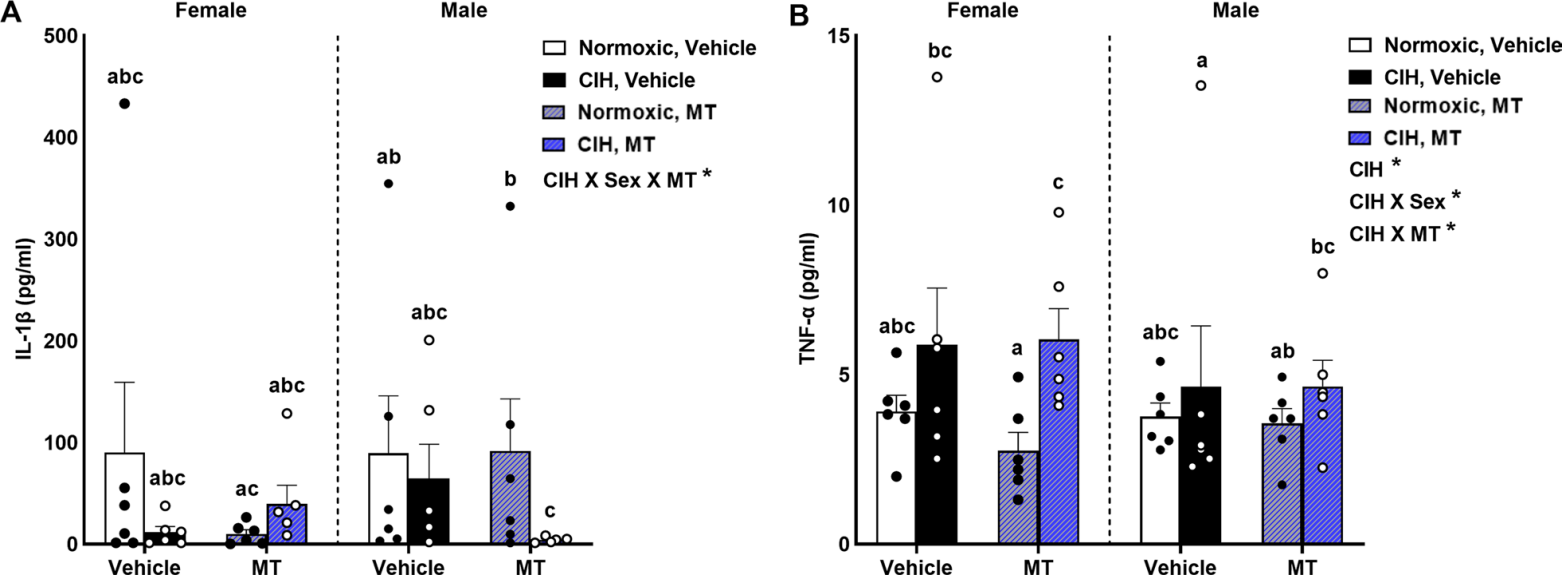
Additional pro-inflammatory cytokines. Pro-inflammatory cytokines were increased in both males and females by CIH. MT only lowered IL-1β levels in males (**A**). Normoxic MT males had higher levels of IL-1β levels than normoxic MT females (**A**). CIH increased TNF-α levels in females, particularly in MT females (**B**). Data for inflammatory cytokines was log transformed for analysis. Raw values are shown and error bars denote mean ± S.E.M. Analyzed by 3-way ANOVA with Fisher’s LSD multiple comparisons tests. ANOVA significance indicated by: ***p < 0.001; Post-hoc significance indicated by unique letters (p ≤ 0.05). Significant effect observed (**A**): CIH X Sex X MT (F_1, 39_ = 5.558; p = 0.024; η^2^ = 0.111). Significant effects observed (B): CIH (F_1, 39_ = 5.726; p = 0.022; η^2^ = 0.097); CIH X Sex (F_1, 39_ = 5.968; p = 0.019; η^2^ = 0.101); CIH X MT (F_1, 39_ = 5.203; p = 0.028; η^2^ = 0.088). *CIH* chronic intermittent hypoxia, *MT* MitoTEMPOL

Circulating IL-1β levels were dependent on CIH, sex, and inhibition of mtOS with MT (p = 0.024; Fig. 2A). This effect was specifically in males, as inhibition of mtOS lowered IL-1β levels only in CIH exposed males (p ≤ 0.05). Normoxic MT males had higher levels of IL-1β than normoxic MT females (p ≤ 0.05). We observed a significant effect of CIH (p = 0.022), and multiple interactions between CIH and sex (p = 0.019) and CIH and MT (p = 0.028) in levels of TNF-α (Fig. 2B). Specifically, CIH increased circulating TNF-α levels in females, but not males, and had the greatest effect in MT females (p ≤ 0.05).

### CIH did not affect circulating steroid hormones

**Fig. 3:**
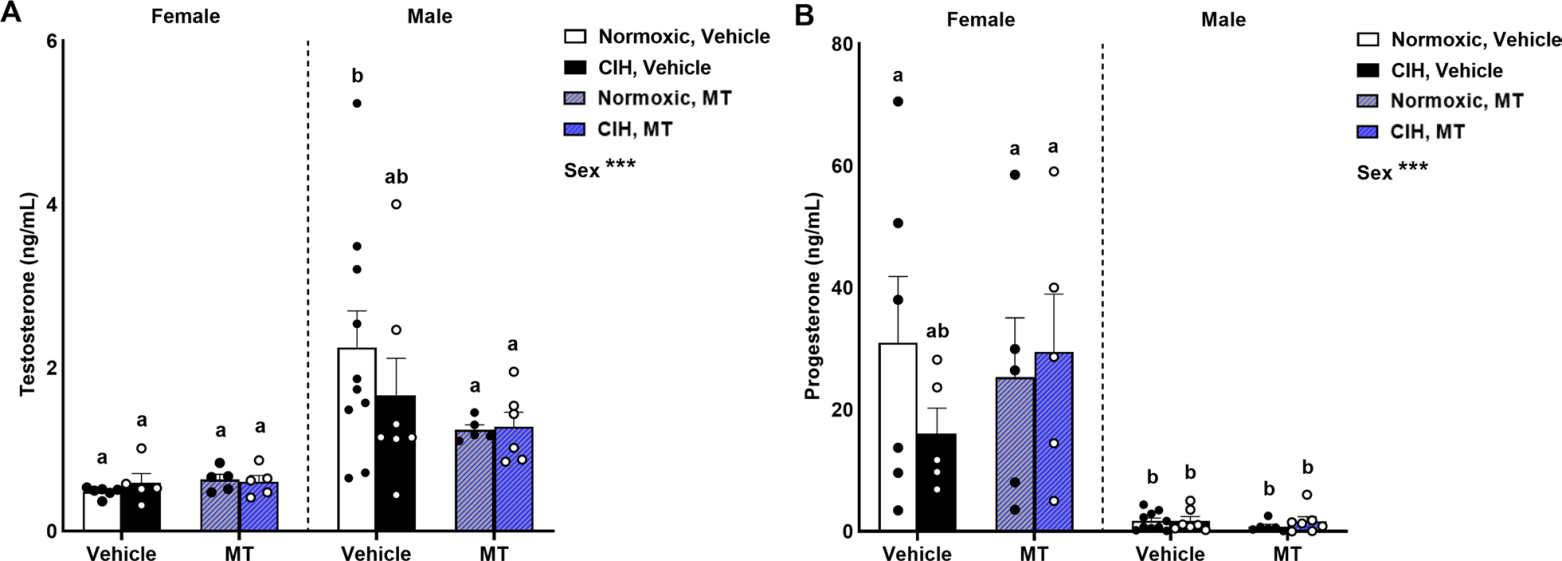
Circulating sex steroid hormones. Male rats had higher levels of circulating testosterone than females (**A**). Female rats had higher levels of progesterone than males (**B**). Raw values are shown and error bars denote mean ± S.E.M. Analyzed by 3-way ANOVA with Fisher’s LSD multiple comparisons tests. ANOVA significance indicated by: ***p < 0.001; Post-hoc significance indicated by unique letters (p ≤ 0.05). Significant effect observed (**A**): Sex (F_1, 41_ = 18.173; p < 0.001; η^2^ = 0.280). Significant effect observed (**B**): Sex (F_1, 43_ = 39.238; p < 0.001; η^2^ = 0.452). *CIH* chronic intermittent hypoxia, *MT* MitoTEMPOL

We observed that male rats had higher circulating testosterone levels than females (p < 0.001; Fig. 3A), regardless of CIH or inhibition of mtOS with MT. Female rats were also observed to have higher circulating progesterone levels than males (p < 0.001; Fig. 3B), regardless of CIH or inhibition of mtOS with MT. No effects of CIH, sex, or mtOS inhibition on circulating estradiol or corticosterone levels was observed (Additional file 1: Table S1).

### CIH increased circulating oxidative stress in males that was reduced by blocking mitochondrial oxidative stress

**Fig. 4:**
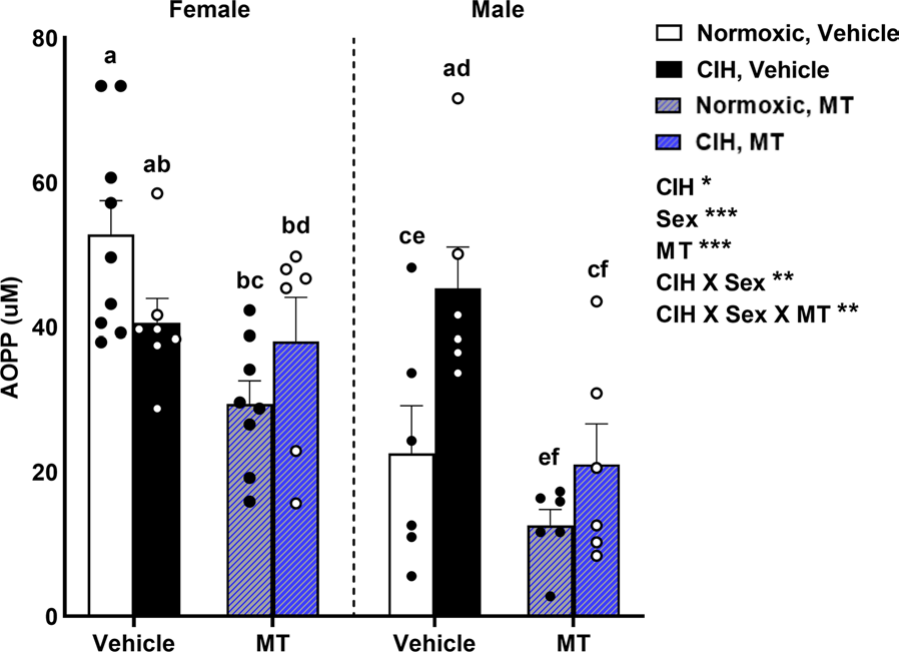
Circulating oxidative stress. AOPP concentrations were dependent on CIH, sex, and MT. In female rats, normoxic females had the highest AOPP concentrations, and these were reduced by MT. CIH also reduced AOPP concentrations in vehicle females, but did not affect AOPP concentrations in MT females. AOPP concentrations were higher in females than males. In vehicle males, CIH increased AOPP concentrations, and this effect was blocked by MT. Raw values are shown and error bars denote mean ± S.E.M. Analyzed by 3-way ANOVA with Fisher’s LSD multiple comparisons tests. ANOVA significance indicated by: *p ≤ 0.05; **p ≤ 0.01; ***p < 0.001; Post-hoc significance indicated by unique letters (p ≤ 0.05). Significant effects observed: CIH (F_1, 46_ = 4.071; p = 0.049; η^2^ = 0.040); Sex (F_1, 46_ = 18.743; p < 0.001; η^2^ = 0.184); MT (F_1, 46_ = 19.286; p < 0.001; η^2^ = 0.190); CIH X Sex (F_1, 46_ = 6.432; p = 0.015; η^2^ = 0.063); CIH X Sex X MT (F_1, 46_ = 6.590; p = 0.014; η^2^ = 0.065). *AOPP* advanced oxidation protein products, *CIH* chronic intermittent hypoxia, *MT* MitoTEMPOL

We observed that circulating OS (plasma oxidized proteins; AOPP) was dependent on CIH, sex, and inhibition of mtOS with MT: main effects of CIH (p = 0.049), sex (p < 0.001), and MT were observed on AOPP (p < 0.001; Fig. 4). We also observed significant interactions between CIH and sex (p = 0.015) and between CIH, sex, and MT on AOPP (p = 0.014; Fig. 4). Overall, AOPP concentrations were higher in females than males (p ≤ 0.05). Normoxic females had the highest AOPP concentrations, which were reduced by MT (p ≤ 0.05). Normoxic MT males had the lowest AOPP concentrations, which were significantly lower than all females (p ≤ 0.05). Exposure to CIH exhibited sex-dependent effects. CIH reduced AOPP concentrations in females (p ≤ 0.05), and inhibition of mtOS using MT only reduced AOPP concentrations in normoxic females with no effect in CIH females. In contrast to females, CIH increased AOPP concentrations in vehicle males (p ≤ 0.05), and this CIH-induced OS was blocked by inhibiting mtOS with MT. AOPP concentrations in CIH vehicle males were increased to levels observed in females (except MT normoxic females) (p ≤ 0.05).

### CIH impaired recollective memory in male and female rats, which was unaffected by inhibiting mitochondrial oxidative stress

**Fig. 5:**
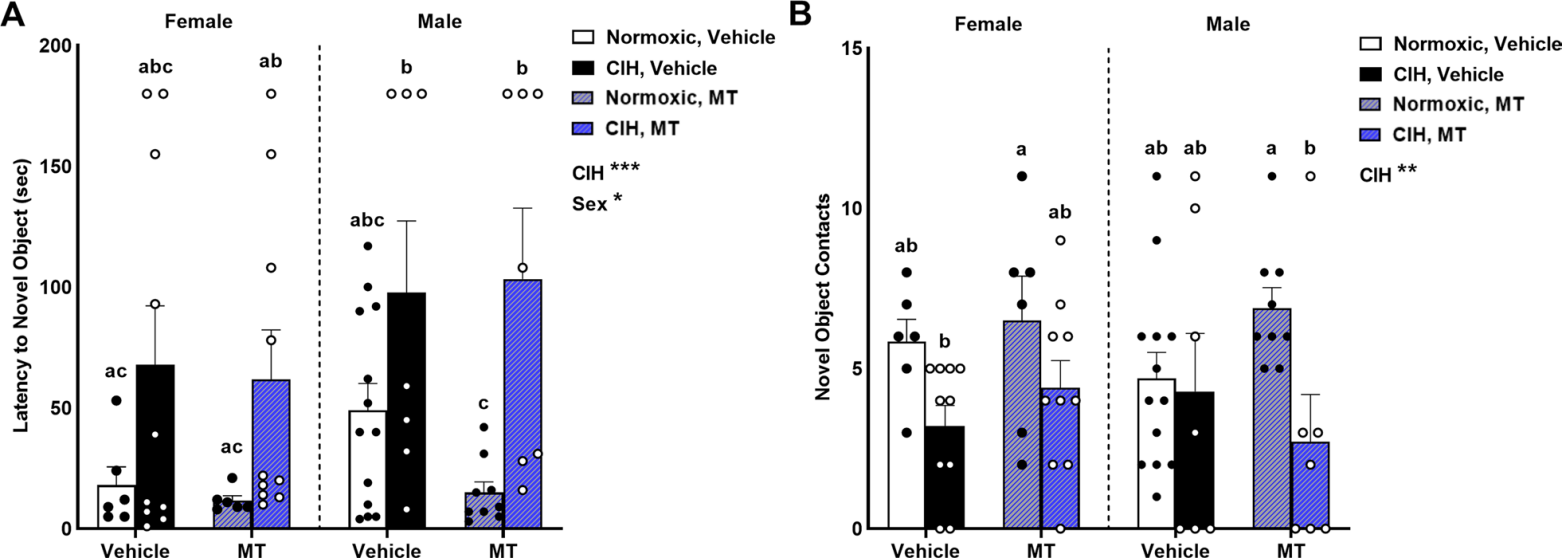
Recollective memory. CIH impaired recollective memory in the novel object recognition task by increasing latency to the novel object (**A**) and decreasing novel object contacts (**B**). Female rats had shorter latency to the novel object than males (F_1, 60_ = 4.386; p = 0.040; η^2^ = 0.053; **A**). No effect of sex was observed on novel object contacts (B). No effect of MT was observed on recollective memory (**A, B**). Data for novel object latency was log transformed for analysis. Raw values are shown and error bars denote mean ± S.E.M. Analyzed by 3-way ANOVA with Fisher’s LSD multiple comparisons tests. ANOVA significance indicated by: *p ≤ 0.05; **p ≤ 0.01; ***p < 0.001; Post-hoc significance indicated by unique letters (p ≤ 0.05). Significant effects observed (**A**): CIH (F_1, 60_ = 14.011; p < 0.001; η^2^ = 0.141); Sex (F_1, 60_ = 4.386; p = 0.040; η^2^ = 0.053). Significant effects observed (**B**): CIH (F_1, 60_ = 9.664; p = 0.003; η^2^ = 0.131). *CIH* chronic intermittent hypoxia, *MT* MitoTEMPOL

Recollective memory was measured in the novel object recognition task. CIH significantly increased latency to the novel object (p < 0.001; Fig. 5A), and this memory impairment was unaffected by blocking mtOS with MT. Females also had shorter latency to the novel object than males (p = 0.040; Fig. 5A), especially in normoxic females compared to CIH males (p ≤ 0.05). In addition to increasing latency to the novel object, CIH significantly decreased novel object contacts (p = 0.003; Fig. 5B) with the greatest effect seen in vehicle females and MT males (p ≤ 0.05). No effects of sex or inhibiting mtOS with MT were observed on novel object contacts (Fig. 5B).

We also examined spatial learning during the Morris water maze. No effects of CIH, sex, or MT were observed on LI (Additional file 1: Table S2). Next, we examined spatial memory by performing a probe trial test on the final day of the Morris water maze. No effects of CIH, sex or inhibition of mtOS with MT were observed on latency to the probe target or pathlength to the probe target in the Morris water maze (Additional file 1: Table S2).

### CIH impaired fine motor function in female rats that was reduced by blocking mitochondrial oxidative stress

**Fig. 6:**
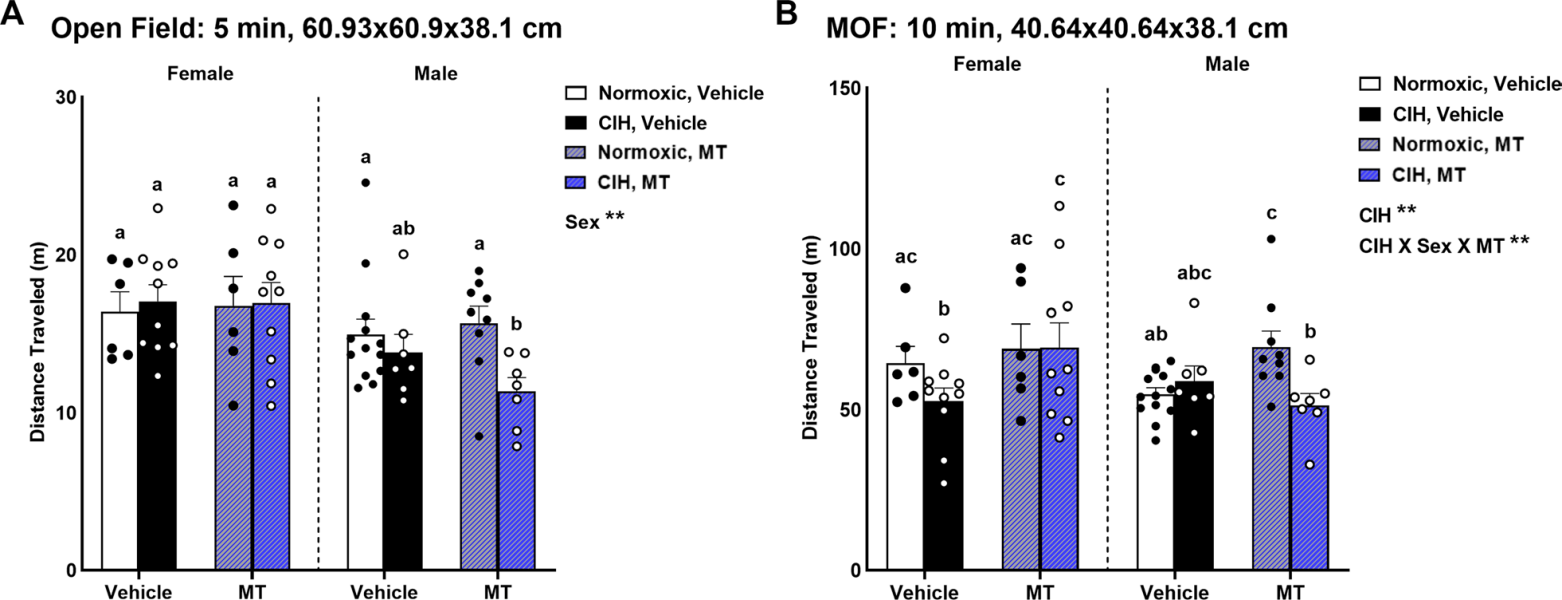
Motor function. Female rats traveled farther in the open field than males (**A**). No effect of CIH or MT was observed in the open field (**A**). Distance traveled in the MOF was dependent on CIH, sex, and MT (**B**). CIH decreased MOF distance traveled in vehicle females, which was prevented by MT (**B**). CIH MT females had elevated MOF distance traveled compared to both normoxic vehicle males, and CIH MT males (**B**). In normoxic males, MT increased MOF distance traveled, but this effect was not seen in CIH males (**B**). Data for MOF distance traveled was log transformed for analysis. Raw values are shown and error bars denote mean ± S.E.M. Analyzed by 3-way ANOVA with Fisher’s LSD multiple comparisons tests. ANOVA significance indicated by: **p ≤ 0.01; Post-hoc significance indicated by unique letters (p ≤ 0.05). Significant effect observed (**A**): Sex (F_1, 60_ = 10.598; p = 0.002; η^2^ = 0.136). Significant effects observed (**B**): CIH (F_1, 64_ = 4.706; p = 0.034; η^2^ = 0.058); CIH X Sex X MT (F_1, 64_ = 6.683; p = 0.012; η^2^ = 0.083). *CIH* chronic intermittent hypoxia, *MOF* modified open field, *MT* MitoTEMPOL

To examine gross motor function, we used a large novel open field (60.96 × 60.9 × 38.1 cm). We observed that female rats traveled farther than male rats (p = 0.002; Fig. 6A), particularly compared to CIH males with MT treatment (p ≤ 0.05). No effects of CIH or mtOS inhibition with MT were observed on distance traveled in the open field (Fig. 6A).

Next, we examined fine motor function using a novel modified open field (MOF; 40.64 × 40.64 × 38.1 cm) in which a wire mesh platform elevated 2 cm above the floor was placed in the arena. We observed a significant effect of CIH on MOF distance traveled (p = 0.034; Fig. 6B). In addition, we observed a significant interaction between CIH, sex, and mtOS with MT on distance traveled in the MOF (p = 0.012; Fig. 6B). Specifically, CIH reduced MOF distance traveled in vehicle females, which was blocked by mtOS inhibition using MT (p ≤ 0.05). CIH did not impact fine motor function (distance traveled) in males. However, inhibition of mtOS did increase fine motor function in normoxic males (p ≤ 0.05). No effects of CIH, sex, or MT, were observed on other measures of fine motor function—assisted rears, unassisted rears, total rears, or footfalls in a modified open field (Additional file 1: Table S3).

### No effects of sex or CIH on anxiety-like behavior in an open field

**Fig. 7:**
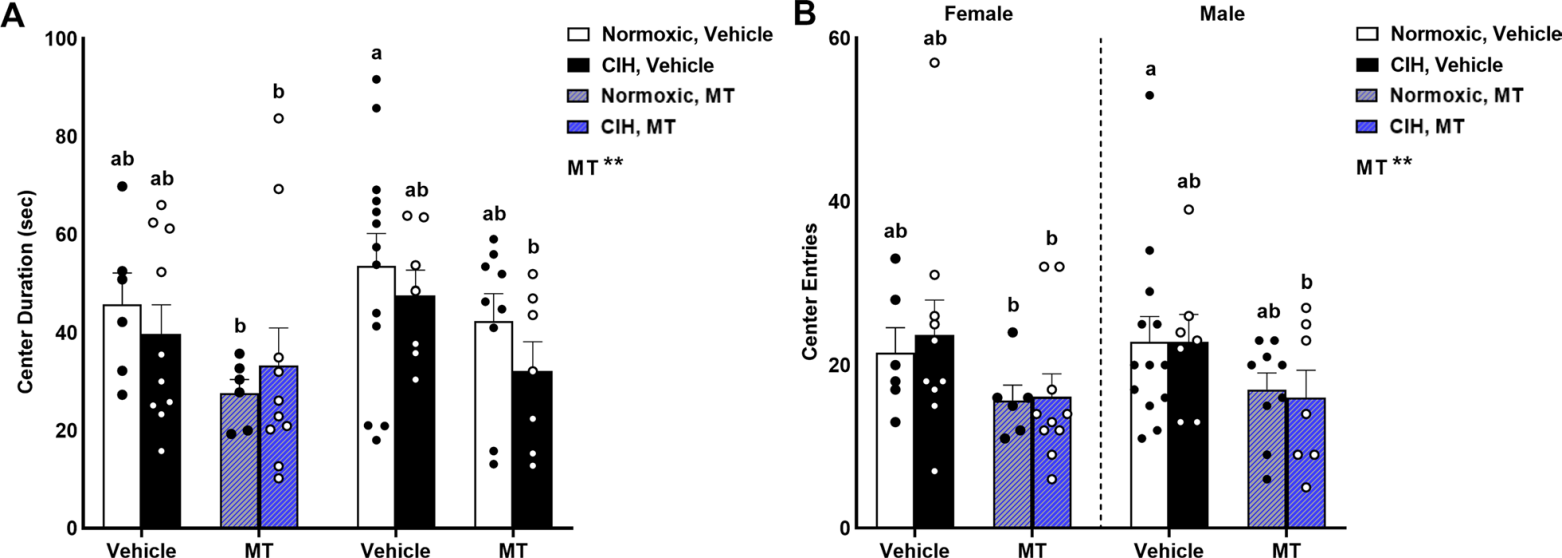
Anxiety-like behavior. Inhibiting mitochondrial oxidative stress reduced center duration (**A**) and center entries (**B**) in an open field arena (60.96 × 60.9 × 38.1 cm). No effects of CIH or sex were observed on center entries or center duration. Raw values are shown and error bars denote mean ± S.E.M. Analyzed by 3-way ANOVA with Fisher’s LSD multiple comparisons tests. ANOVA significance indicated by: **p ≤ 0.01; Post-hoc significance indicated by unique letters (p ≤ 0.05). Significant effect observed (**A**): MT (F_1, 60_ = 7.434; p = 0.008; η^2^ = 0.110). Significant effect observed (B): MT (F_1, 60_ = 7.301; p = 0.009; η^2^ = 0.101). *CIH* chronic intermittent hypoxia, *MT* MitoTEMPOL

We examined anxiety-like behaviors using a large novel open field (60.96 × 60.9 × 38.1 cm) by quantifying the frequency and duration of entries within the center of the large open field. No effects of CIH or sex were observed on anxiety-like behaviors—center duration or center entries (Fig. 7). However, inhibition of mtOS did decrease open field anxiety-like behaviors, as evidenced by decreased center duration (p = 0.009; Fig. 7A) and center entries (p = 0.008; Fig. 7B).

### CIH increased compulsive behavior in male rats that was prevented by blocking mitochondrial oxidative stress

**Fig. 8:**
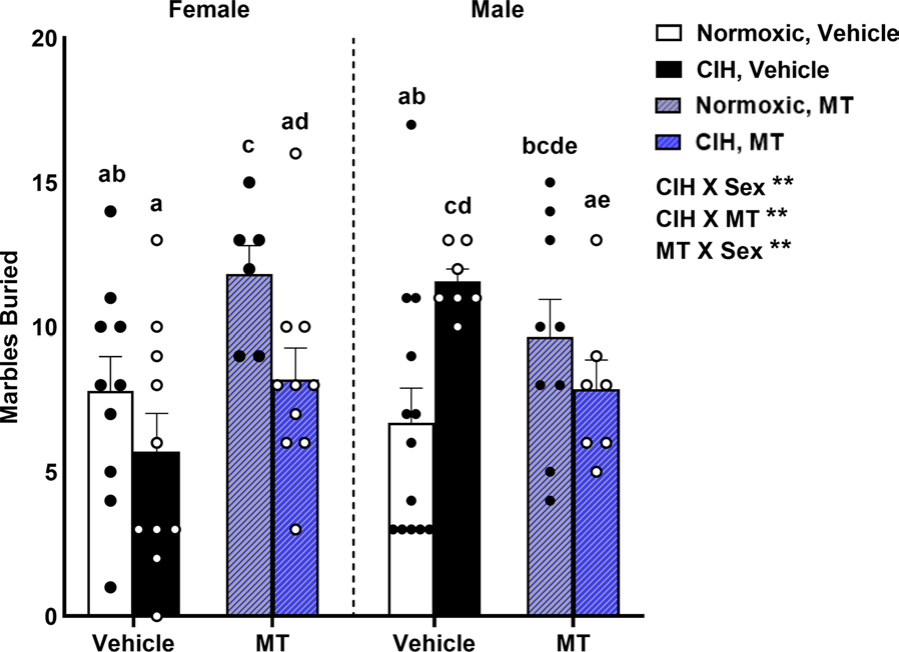
Compulsive behaviors. CIH increased marble burying in vehicle male rats, but not in vehicle females. MT prevented CIH from increasing marbles buried in males. In MT females, CIH decreased marble burying, with no effect in MT males. MT increased marble burying in normoxic females, but not in normoxic males. Raw values are shown and error bars denote mean ± S.E.M. Analyzed by 3-way ANOVA with Fisher’s LSD multiple comparisons tests. ANOVA significance indicated by: **p ≤ 0.01; Post-hoc significance indicated by unique letters (p ≤ 0.05). Significant effects observed: CIH X Sex (F_1, 64_ = 6.499; p = 0.013; η^2^ = 0.075); CIH X MT (F_1, 64_ = 5.669; p = 0.020; η^2^ = 0.065); MT X Sex (F_1, 64_ = 4.437; p = 0.039; η^2^ = 0.051). *CIH* chronic intermittent hypoxia, *MT* MitoTEMPOL

We examined the effects of CIH, sex, and mtOS inhibition on compulsivity using the marble burying task. We observed multiple significant interactions on marble burying between: (1) CIH and sex (p = 0.013), (2) CIH and MT (p = 0.020), and (3) MT and sex (p = 0.039; Fig. 8). CIH increased marble burying in vehicle males (p ≤ 0.05), which was prevented by blocking mtOS with MT. However, CIH did not affect marble burying in vehicle females. Inhibiting mtOS in normoxic females increased marble burying (p ≤ 0.05).

## Discussion

The major findings of this study are 1) 14-day CIH-induced OS and inflammation are sex- and mtOS-dependent, whereas 2) 14-day CIH-induced behavioral changes are dependent on multiple factors that include sex and mtOS. Specifically, we found that CIH increased inflammation and fine motor dysfunction in female rats, which was blocked by inhibiting mtOS. Conversely, CIH increased circulating OS and compulsive behavior in males, which was blocked by inhibiting mtOS. However, we observed no sex differences in CIH-induced recollective memory impairment. Both male and female rats exposed to CIH performed worse than normoxic controls in the novel object recognition task, which was unaffected by inhibition of mtOS.

This study is the first study to examine the effects of CIH on inflammatory cytokines in females, along with the first study to compare sex differences in CIH-induced inflammatory cytokines. We observed that CIH increased circulating IL-6 levels, decreased IL-10 levels, and increased IL-6/IL-10 ratio in females. IL-6 is a pleiotrophic cytokine that is associated with many inflammatory diseases [98], whereas IL-10 is an anti-inflammatory cytokine that is linked with downregulating the effects of IL-6 in the immune response [101, 104]. The IL-6/IL-10 ratio indicates the status of pro-inflammatory and anti-inflammatory processes in circulation [102, 104]. Few studies have examined sex differences in basal plasma cytokine levels; however, female rats typically have higher levels of IL-6 and TNF-α than males [105, 106]. In our study, we observed no sex differences in the level of any cytokines, which is consistent with other studies examining sex and cytokine levels in plasma [105, 106].

Although prior studies examining the impact of CIH on the immune system have only examined male rodents, they observed increased pro-inflammatory cytokines IL-6, TNF-α, and IL-1β in the circulation in response to CIH [53, 54, 107, 108]. In contrast to these studies, our study found that CIH did not impact IL-6, TNF-α, or IL-1β levels in male rats. This negative finding is consistent with our previous publication that showed no effect of CIH on IL-6 levels or TNF-α levels [6]. One possible explanation for this discrepancy in CIH-induced cytokine levels in males could be the CIH protocol. Our studies utilized a CIH protocol with an AHI of 10 (10 hypoxic episodes/hr/over 8 h/day) to model mild sleep apnea in humans [31, 79]. In contrast, other laboratories used different CIH protocols in male rats that range in AHIs of 30–40 with similar CIH protocol duration (8 h/day; 14–21 days) [54, 107, 108], which is consistent with severe sleep apnea in humans [31]. This could indicate that the inflammatory response to CIH in males is dependent on AHI severity. Though CIH primarily increased pro-inflammatory cytokines in females, MT was observed to reduce pro-inflammatory cytokines in both sexes and stimulated anti-inflammatory cytokines in females. These results support previous findings that MT and similar compounds can reduce pathogenic inflammatory states in response to injury or illness [109, 110].

Similar to our cytokine data, we observed sex differences in circulating oxidized protein levels (AOPP). Consistent with prior reports [6, 55, 56], we found that CIH increased AOPP concentrations in male rats. However, this is the first study to show that CIH-elevated AOPP in male rats was mediated by mtOS. In contrast to male rats, CIH did not increase AOPP in female rats, which is consistent with other studies showing no effects of CIH on circulating OS measures [55, 111]. We also found sex differences in basal circulating OS, in which normoxic female rats had higher OS levels than male rats. This finding is consistent with clinical data wherein women frequently exhibit higher circulating OS than men [41–43].

This study is the first to examine the effects of CIH exposure on circulating steroid hormones in male and female rats. CIH did not affect circulating steroid hormone levels in any measured hormone, regardless of sex. As expected, testosterone and progesterone levels were dependent on sex, whereas estradiol and corticosterone were not different between sexes. These results are consistent with previous studies measuring circulating testosterone, progesterone, and estradiol levels in rats [78, 112–118]. However, the effects of sex on corticosterone are less clear, wherein prior studies have shown either higher corticosterone levels in females [119–122] or no sex differences [78]. Previous studies have found testosterone to be either reduced by CIH exposure in male rats [65] or unaffected by CIH exposure in male or female rats [123]. CIH exposure was not observed to affect estradiol levels in either male or female rats [123]. In male rats, corticosterone has been observed to be either increased by CIH [79, 124] or no effect of CIH was observed [65, 79]. Prior studies have shown strain differences in response to CIH on steroid hormone levels [79]. Strain differences could explain why CIH did not affect steroid hormones in our rats, and why our findings are consistent with previous studies that used Sprague Dawley rats in their experiments [79, 123].

This study is the first to examine sex differences in CIH-induced behavior displayed by adult rats. We observed distinct sex differences in CIH-induced fine motor function impairment and compulsivity but not in CIH-induced recollective memory impairments. CIH reduced fine motor function only in females, which was blocked by inhibiting mtOS. Since inhibiting mtOS decreased CIH-induced inflammation in females, we propose that a mtOS-inflammation feed-forward mechanism is involved in CIH-induced fine motor impairment in females. Indeed, inflammation has been observed to impair motor behaviors [125, 126], which may be related to CIH-induced neuroinflammation in motor behavior-associated brain regions in rats [6, 107]. 

This is the first study to examine the impact of CIH on marble burying. We observed that CIH increased compulsive behavior in male rats but not female rats. Further, inhibition of mtOS blocked CIH-induced compulsivity in males. Since inhibiting mtOS decreased CIH-induced OS in our males and CIH increases OS damage to brain regions [6, 63, 127] associated with marble burying behavior (e.g., hippocampus, orbitofrontal cortex, striatum) [84, 128, 129], mtOS may be one of the primary mechanisms in CIH-induced compulsivity in male rats.

We did not observe any sex differences in CIH-induced recollective memory impairment. Consistent with prior studies showing CIH-induced impairments in recollective memory in male rodents [57–59], we also found that CIH impaired recollective memory in male rats. CIH also decreased recollective memory in females, and our study is the first to examine these relationships. Interestingly, inhibiting mtOS did not prevent the effects of CIH on recollective memory. This indicates that a mtOS-independent mechanism induced by CIH is impairing recollective memory in both sexes. Although no prior studies have examined the effect of CIH on recollective memory in females, studies have examined sex differences in recollective memory in response to neurotoxins. These findings on sex differences in recollective memory were equivocal, in which some studies show no sex differences in recollective memory impairment [130, 131] or only males were impaired and not females [130, 132].

CIH had no effects on spatial learning and memory or anxiety-like behavior in either male or female rats. Our findings are consistent with prior reports demonstrating CIH did not impact spatial learning and memory in gonadally intact female mice [66]. However, in contrast to prior studies showing CIH impairment of spatial learning and memory in male rats [53, 60–62], we did not observe any effects of CIH on spatial learning and memory in males. Studies routinely report males exposed to CIH exhibit increased anxiety-like behaviors [58, 63, 64], which was not observed in this study. It should be noted that these prior studies in which CIH induced spatial learning and memory impairments and anxiety-like behaviors were conducted with different CIH protocols (AHI ranged from 40 to 60) [53, 58, 60–64] compared to our CIH protocol (AHI = 10). Indeed, a study using a CIH protocol with an AHI of 20 found no effect of CIH on spatial learning and memory in male rats [133], and another study using an AHI of 15 found no effects on anxiety-like behavior in the open field in male and female mice [66]. These findings indicate that CIH protocols with > 20 AHI is necessary to induce spatial learning and memory impairments and anxiety-like behaviors.

We also observed sex differences in learning and motor behaviors exhibited by our normoxic rats. Consistent to prior reports showing females exhibit greater recollective memory than males [134–136], we also found that females exhibited better recollective memory (shorter latency to novel object) than males. Prior studies have typically shown either male biases [78, 134, 137] or no sex differences in spatial learning via Morris water maze [137, 138]. Neither LI scores nor latency to platform during the probe trial detected significant sex differences, which are consistent with previous studies [137, 138]. Consistent with prior reports, female rats showed greater locomotor activity than male rats in a large novel open field arena (60.96 × 60.9 × 38.1 cm) and no sex differences in a smaller novel open field (40.64 × 40.64 × 38.1 cm) [52, 139]. Unlike these findings, we did not observe any sex differences in marble burying behavior in normoxic rats. The data on sex differences in marble burying is equivocal, with some groups finding either no sex differences [140, 141], a female bias [142], or a male bias [78, 143].

### Limitations

Although our study has many strengths ranging from examining a broad spectrum of circulating markers (inflammation, OS, steroid hormones) and multiple behavioral domains, there are some limitations. We did not assess estrous cycle in our female rats. It is probable that the variability in the female data is related to estrous cycle, as many of the outcomes measured in our study could be influenced by estrogen status [144–146]. However, we did not want to introduce a stress response by conducting vaginal smears [147], nor introduce a variable that could not be conducted on the male rats. Notably, we did not observe any effects of CIH on estradiol or progesterone levels in females, which could indicate that estrous cycling was unaffected by CIH. Additionally, we did not determine the brain concentrations of MT. However, our data showing behavior responses to MT, data showing that MT has similar membrane permeability to TEMPOL [75], and data that TEMPOL penetrates the blood–brain-barrier [74] indicates that MT has actions at the level of the brain.

### Perspectives and significance

Our data indicates that mild CIH (AHI = 10) can have significant impacts on multiple domains, such as circulating OS and inflammation, recollective memory, fine motor function and compulsivity. Furthermore, most of these CIH effects are sex- and mtOS-dependent with the exception of recollective memory impairment. This is of concern, as patients with sleep apnea with AHIs < 15 are classified as having mild OSA [14, 16, 18], and therefore may not receive treatment due to fewer reported quality of life impairments [4, 148, 149]. This lack of treatment for mild OSA can affect women more than men, as women are more likely to present with mild OSA or be underdiagnosed with OSA [4, 5]. We also show that many of the sex-dependent effects of CIH are mediated through mtOS, which may be a plausible target for therapeutics aimed at 15–40% of OSA patients that are unable to be effectively treated with CPAP machines [26–28].

### Supplementary Information

**Additional file 1: Table S1.** Circulating steroid hormones. Plasma steroid hormones were analyzed by high throughput multiplex. All values presented as mean ± SD. Analyzed by 3-way ANOVA with Fisher’s LSD multiple comparisons tests, n = 5–10/group. *CIH: Chronic intermittent hypoxia; MT: MitoTEMPOL.* **Table S2. **Morris water maze results. Latency and pathlength (cm) for rats to find the target location during the probe trial. Learning index was generated as the sum total of the average latency to the target of all trials in blocked means for each training day. All values presented as mean ± SD. Analyzed by 3-way ANOVA with Fisher’s LSD multiple comparisons tests, n = 5–8/group. *CIH: Chronic intermittent hypoxia; MT: MitoTEMPOL.* **Table S3.** Fine motor behavior in modified open field. Rearing behavior and footfalls in an open field arena (40.64 × 40.64 × 38.1 cm) modified with a 2 cm elevated wire mesh platform. Rears were categorized as assisted if the rat used forelimbs to brace against side of open field arena. All values presented as mean ± SD. Data for unassisted rears was square root transformed for analysis. Analyzed by 3-way ANOVA with Fisher’s LSD multiple comparisons tests, n = 6–13/group. *CIH: Chronic intermittent hypoxia; MT: MitoTEMPOL.*

## Data Availability

The datasets used and/or analyzed during the current study are available from the corresponding author upon reasonable request.
